# Supplementary figures and images for: Assessment of anesthesia capacity for children in Somaliland
Source: PLOS Glob Public Health. 2024 Aug 28;4(8):e0003650. doi: 10.1371/journal.pgph.0003650 (PMC11356410; doi:10.1371/journal.pgph.0003650)

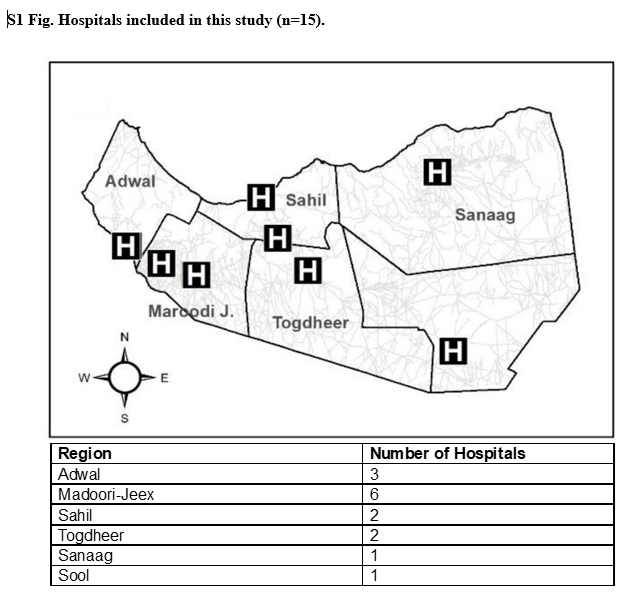

Supplement: S1 Fig — (PNG) [file pgph.0003650.s001.png]

**S2 File. Hospital capacity assessment.**


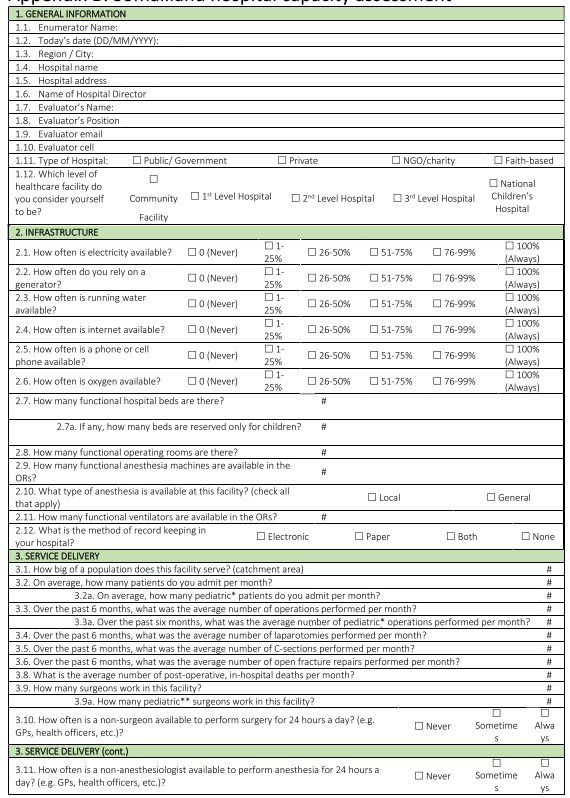


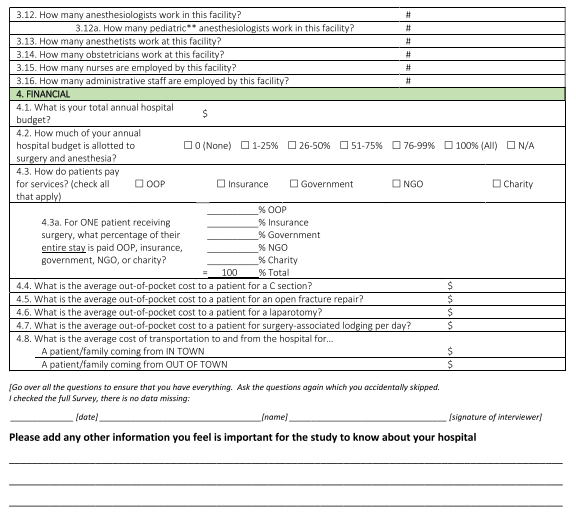

Supplement: S1 Text — (DOCX) [file pgph.0003650.s002.docx]

**S3 File. Surgical record tool.**


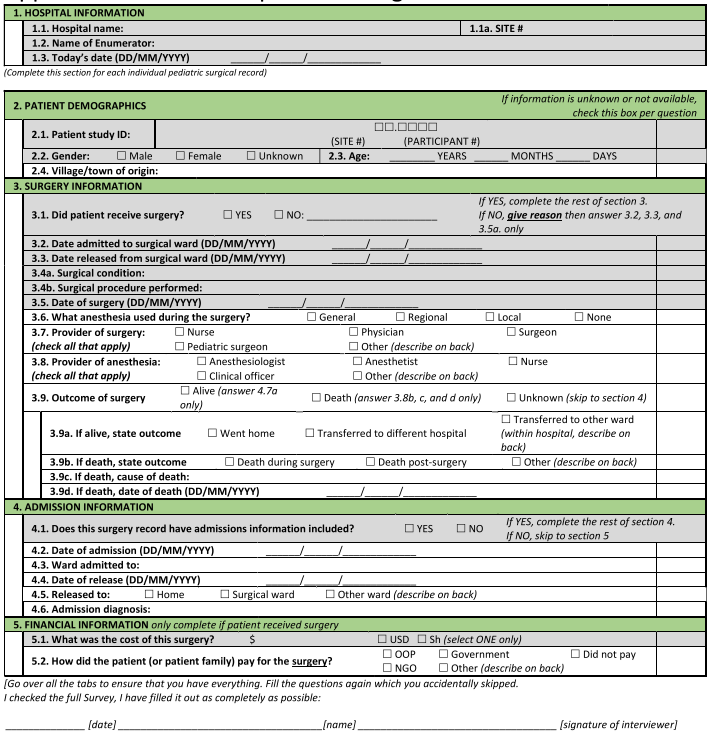

Supplement: S2 Text — (DOCX) [file pgph.0003650.s003.docx]
