# Supplementary material for: Assessment of anesthesia capacity for children in Somaliland
Source: PLOS Glob Public Health. 2024 Aug 28;4(8):e0003650. doi: 10.1371/journal.pgph.0003650 (PMC11356410; doi:10.1371/journal.pgph.0003650)
Supplement: Attachment — Submitted filename: Response to reviewers_Anesth SL_PLOS GPH.docx [file pgph.0003650.s006.docx]

PGPH-D-23-02129

**Assessment of Anesthesia Capacity for Children in Somaliland**

PLOS Global Public Health

Dear Dr. M. Dylan Bould,

We would like to thank you for the opportunity to revise our manuscript. We found the critiques and suggestions to be very helpful in making this publication as strong as it can be, as we believe it could be a significant contribution to the growing body of literature on the importance of anesthesia capacity in Somaliland.

We have submitted a marked-up copy and an unmarked version of the revised manuscript. A rebuttal letter addressing all critiques and suggestions is also included. We look forward to hearing from you. Thank you for your time and consideration.

Sincerely,

The Authors.

In this document, we have organized the comments by reviewer (Academic editor, Reviewer 1, Reviewer 2) as follows:

**Bolded text**

are the original comments from the reviewers

**REPLY**

include our responses

**Academic Editor:**

 **1. Please include a complete copy of PLOS’ questionnaire on inclusivity in global research in your revised manuscript. Our policy for research in this area aims to improve transparency in the reporting of research performed outside of researchers’ own country or community. The policy applies to researchers who have travelled to a different country to conduct research, research with Indigenous populations or their lands, and research on cultural artefacts. The questionnaire can also be requested at the journal’s discretion for any other submissions, even if these conditions are not met. Please find more information on the policy and a link to download a blank copy of the questionnaire here: https://journals.plos.org/globalpublichealth/s/best-practices-in-research-reporting. Please upload a completed version of your questionnaire as Supporting Information when you resubmit your manuscript.**

**REPLY**

We thank the editor for this suggestion. We have included the PLOS questionnaire on inclusivity in global research.

**2. You need to include the actual survey tool that you used in this study as an appendix.**

**REPLY**

We thank the editor for this question. We have included the mentioned survey tools as appendices.

**3. You need to explain the process for adapting existing tools and the justification for this.**

**REPLY**

We thank the editor for this question. This process can be found in the original cited study (citation #16).

**4. How did you identify the ''one anesthesia representative" interviewed for each site?**

**REPLY**

We thank the editor for this question. The research coordinator asked permission from the lead clinician or administrator at each hospital (preferably the hospital director) for participation in the study and presented the study’s letter of permission from the Ministry of Health (MoH). Then, the lead clinician identified and appointed a surgical/anesthesia representative to be interviewed using the one-page hospital capacity survey. This detail can also be found in citation #16.

**5. It is important to clarify to what extent the data overlaps with the previous paper https://pubmed.ncbi.nlm.nih.gov/31297580/**

**REPLY**

We thank the editor for this question. While the data collection tools used in both articles are the same, our previous publication focuses on the overall availability of surgical care in Somaliland, while our current study aims to focus on a detailed description of anesthesia care. The point where both manuscripts overlap is in reporting two of the four main indicators in anesthesia capacity, the overall number of anesthesia providers and operating rooms. However, Table 2 offers a more granular look at anesthesia capacity, including new information like type of anesthesia (general and regional) and type of anesthesia provider (anesthesiologist and NPAP). Finally, we would like to note that some overlap was needed to understand the full context of anesthesia capacity in this study.

**6. The results presented are really quite superficial and certainly do not include all the factors that need to be considered when assessing capacity for providing pediatric anesthesia. Do you have other data from your tool that can be presented as supplementary materials.**

**REPLY**

We thank the editor for this suggestion. I do wish we had more data to present for anesthesia! However, this is all we have at the time.

**7. Please avoid the unreflective use of the term 'low and middle income countries/LMIC" - see https://www.ncbi.nlm.nih.gov/pmc/articles/PMC9185389/ - at best it is terminology that dichotomizes the world, including a very heterogenous group of countries (and contexts within countries) within a single category.**

**REPLY**

We thank the editor for this excellent suggestion. We agree and we have revised the use of LMICs across the manuscript.

**Reviewer 1:**

**Thank you for the opportunity to review this paper and apologies for the slow review. I have read with interest the manuscript entitled, ‘Assessment of Anesthesia Capacity for Children in Somaliland’. The article is written with the purpose of describing the anesthesia capacity for children, including workforce, infrastructure, and equipment across Somaliland. This evaluation is intended to serve as a foundation for making recommendations to scale up pediatric anesthesia capacity in Somaliland. The manuscript is generally well-written and organized, making it easy to follow the flow of ideas. Addressing the suggested revisions outlined below would further enhance the quality and impact of the study.**

**1. Abstract: The article describes the anesthesia capacity for children in Somaliland; however, the abstract seems to suggest the aim is to describe anesthesia capacity in Somaliland (line 43). It would do well to stick to the aim as stated in the title. This makes it a unique paper and a great addition to the literature on anesthesia capacity for children in LMICs.**

**REPLY**

We thank the reviewer for this suggestion. We have updated the abstract accordingly.

**2. Introduction: The WFSA Global Anesthesia workforce study referred to in (line 82) has been updated. It might give us a good representation of the state compared to the global status. The Global Anesthesia Workforce Survey: Updates and Trends in the Anesthesia Workforce. Anesthesia and Analgesia. https://doi.org/10.1213/ANE.0000000000006836 . This could enrich the context of the study and its relevance to broader anesthesia workforce trends.**

**REPLY**

We thank the reviewer for this suggestion. We have included the updated version of the WFSA Global Anesthesia Workforce Survey in the introduction

**3. Methods (Data Collection): In line 126, you state “previously validated hospital capacity survey” but no reference is provided to this. At least as an attachment (supplementary material) will be useful to make the methods section clearer.**

**REPLY**

We thank the reviewer for this suggestion. We have included the data collection tools as supplementary files in this revision. Further details are also available a few lines below, under the “Hospital capacity survey” and “Pediatric surgical record review” subheadings.

**4. Results: Stating the breakdown of the categories of hospitals will provide more information about access to surgical care rather than lumping them up into one basket of “A total of 15 private, charity, and government hospitals” (line 170). If the majority of these hospitals were private, it would reveal even greater challenges regarding access to surgical care for children.**

**REPLY**

We thank the reviewer for this suggestion. We have updated the mentioned sentence and included a breakdown of the hospitals as follows: “*Of the 15 hospitals, eight were government hospitals, five were private/for-profit hospitals, and two were charity hospitals. Of the 8 government hospitals, one was a national hospital, six were regional hospitals, and one was a district hospital.”*

**5. Were the 7 functional ventilators in (line 182) having the capacity to ventilate neonates? It would be good to state that since you interacted with at least one anesthesia provider from each hospital. It is not uncommon to find ventilators that are only able to ventilate adults and maybe big children (teenagers) in low-income countries. The same applies to anesthesia machines (line 181) because having a functional anesthesia machine is different from having one that can take care of neonates. This would add to the magnitude of the existing challenges with anesthesia capacity. Maybe provide the operational definition of a functional ventilator as per this study.**

**Additionally, were these ventilators part of the anesthesia machines or independent ICU ventilators? Stating this would give us a better description of the current state.**

**REPLY**

We thank the reviewer for this excellent question and comment. We agree that having more detailed information would add to the magnitude of the existing challenges. Unfortunately, we do not have additional information about whether the ventilators and anesthesia machines had the capacity to serve neonates. We have provided an operational definition of a functional ventilator and anesthesia machine. We have also added the lack of this information as a limitation.

**6. (Line 210) Consider providing an age disaggregation of surgical cases (e.g., neonates, 1 month to 12 months, 1 year to 5 years, and 6 years to 12 years) to contextualize anesthesia capacity relative to different age groups.**

**REPLY**

We thank the reviewer for this suggestion. Given this was a capacity survey at each hospital, we do not have the disaggregated data of surgical cases by age group for the children.

**Reviewer 2:**

**1. Abstract – there is no referencing in the abstract but there should be as it brings up WHO-WFSA standard measurement**

**REPLY**

We thank the reviewer for this suggestion. Unfortunately, we cannot cite references in the abstract. However, we have provided all references, including the reference to the WHO-WFSA standard measurement in the full text.

**2. Suggest considering the addition of a map of Somaliland with the regions outlined and major towns denoted – even in an appendix for those who are not familiar with the country to see better.**

**REPLY**

We thank the reviewer for this suggestion. We have provided a map of Somaliland depicting the number of hospitals by region as supplementary material.

**3. Line 73/74. Consider refashioning the sentence especially with removing the double use of specialized in the same sentence. Suggest rearranging final part ‘ a specialized workforce training’ into a slightly easier to digest phrase.**

**REPLY**

We thank the reviewer for this suggestion. We have updated the mentioned sentence as follows: “*Children require specialized healthcare providers and equipment for ventilation and physiologic monitoring*”

**4. Suggest re-reading the article with particular attention paid to spacing at the end of a sentence and referencing e.g. lines 77,82, 92, 108, 156 as a few examples where this isn’t done.**

**REPLY**

We thank the reviewer for this suggestion. We have double-checked the manuscript according to the reviewer’s advice.

**5. Line 107 – lose ‘s’ in millions and consider addition of people after million.**

**REPLY**

We thank the reviewer for this suggestion. We have fixed this typo according to the reviewer's advice.

**6. Line 113 – suggest addition of ‘the’ between finance and workforce.**

**REPLY**

We thank the reviewer for this suggestion. We have fixed this typo according to the reviewer's advice.

**7. Data Collection. States that previous data from interviews with anaesthetic providers between 1st Aug 2017 and 31st Dec 2017. This coincides with the paper referenced 19, which relates to collating data from surgical log books AUGUST 2016 – JULY 2017. It states that there were 1255 operations looked at during this time frame and was analysed. This is the same number as stated in the table and results and I would assume that this is the same data. As such, the interviews with anaesthetic providers about provisions and supplies would have taken place after the data collection of the log books and as such there may be some bias of recall of provisions and supplies for the data collected as a different time. But as the data from August 2016-July 2017 is being used this should be included in the data collection prose. But also it does raise the question of why it has taken a length of time to analyse the anaesthetic component of the data from 7 years ago, when the surgical ones were published in 2020.**

**REPLY**

We thank the reviewer for this clarification question and suggestion. We confirm that surgical record data was collected from 01 August 2016 to 31 July 2017 (this was corrected in the manuscript), while hospital capacity surveys were administered from 01 August 2017 to 31 December 2017.

The original version of this manuscript already included that the current study uses data collected as part of previous studies. The following sentence can be found under the “Data collection” subheading: “*This cross-sectional study is an extension of our previous work on surgical care for children across Somaliland*. (references to previous studies)”

The hospital surveys were administered to a lead local representative at each hospital and depicted the current status of anesthesia capacity at that time. Since the surveys are expected to be filled out with information observed in real-time, we do not expect recall bias to be a limitation of this study. The pandemic greatly affected research output in the group since many of the authors pivoted to direct clinical care or epidemiologic work for COVID-19; hence, the delay in publishing this paper.

**8. There is some cross over of the data in the provision of surgical care for children with reference to paper 19 by the same group referenced during the discussion**

**REPLY**

We thank the reviewer for this suggestion. While the data collection tools used in both articles are the same, our previous publication focuses on the overall availability of surgical care in Somaliland, while our current study aims to focus on a detailed description of anesthesia care. The point where both manuscripts overlap is in reporting two of the four main indicators in anesthesia capacity, the overall number of anesthesia providers and operating rooms. However, Table 2 offers a more granular look at anesthesia capacity, including new information like type of anesthesia (general and regional) and type of anesthesia provider (anesthesiologist and NPAP). Finally, we would like to note that some overlap was needed to understand the full context of anesthesia capacity in this study.

**9. Line 147 + 364– the demographics states children 0-15y only looked at which is fine. It is a curious number however. Is 15y the age in Somaliland of becoming an adult? Or is it 15 years and 364 days?**

**REPLY**

We thank the reviewer for this suggestion. 0-15 years means from birth to the end of 15 years.

**10. Line 150 and table 2. 1255 surgical records of paediatric operations. No mention of any that might have been excluded or proportion of the overall workload.**

**REPLY**

We thank the reviewer for this suggestion. We do not have data on the exclusions or the proportion of the overall workload.

**11. Stats analysis: Line 157 – conduct of anaesthesia was excluded but in line 148 it states inclusion of type of anaesthetic administered and line 161 the type of anaesthetic is included.**

**REPLY**

We thank the reviewer for this clarification question. We have deleted the section where we previously stated that the conduct of anesthesia was excluded.

**12. Results. Table 2. I am confused with the total number of catchment area being 4,000,000. But it is stated calculations are using the upper limit of the catchment areas apart from Maroodi-Jeex (but doesn’t state what’s used for this region). But also adding up each region’s capacity the total number stated at 4,000,000 doesn’t add up to the total. (and in fact Maroodi-Jeex is stated at 83-4,000,000 and if upper limit used would exceed the total considerably). Suggest checking through the table and the mathematical analysis again.**

**REPLY**

We thank the reviewer for this question. There is indeed a mismatch in the numbers regarding the total catchment population and the stratification of the catchment population by region. The total catchment population is based on the total population of Somaliland at the time of the data collection and since the majority of children will have to travel to Hargeisa for surgical care. Table 2 depicts the values reported by the hospitals' representatives. In other words, these are the regional sum of the gross estimates of the catchment population. It is also worth noting that hospital representatives may have taken into account villages from neighboring countries as their catchment population as foreign patients are frequently admitted into hospitals like the Edna Adan University Hospital in Hargeisa.

**13. I am also confused with the data set out regarding anaesthetic procedure cover.**

**It suggests that 150 operations were provided by an anaesthesiolist in M-J and 1 in Sahil. And yet when looking at where the anaesthesiologists are stated to reside. 2 are in M-J and one in Sanang and 0 in Sahil – so this seems strange. A typo or mislaid column or a visiting anaesthetist?**

**REPLY**

We thank the reviewer for this suggestion. It must have been a visiting anasthesiologists to the hospital in Sahil.

**14. Also a question – there are more anaesthetic providers present than there are operations conducted (1273 rather than 1255 procedures) . This would seem to suggest that in some operations, more than one provider is present. This would be useful to highlight if so. In lines 212-3 it states there are anaesthetists present in 12% of operations and NPAP in 88%. If it is that there are anaesthetic providers doubled up in some operations then those percentages are inflated and if not more than one anaesthetic provider present then there is a mismatch in the data.**

**REPLY**

We thank the reviewer for this clarification question. We agree with the reviewer's reasoning that in some operation more than one provider was present. The original version of the manuscript clarifies this in one of the table footnotes as follows: “e Multiple selections available in survey, totals may be over or under hospital specific totals.”

The percentages reported in the mentioned lines 212-3 refer to the percentage that anesthesiologists and NPAP make up with respect to their total. The following calculation demonstrates there is no mismatch in the data:

Total anesthesia providers participating in surgical care = 1273 = 100%

Anesthesiologists = 151 =12%

NPAPS = 1122 = 88%

We have included the sample size (n) after each percentage in the results section to clarify this confusion.

**15. Line 225 It highlights regional discrepancies in delivery of anaesthetic care – but it was only meant to be looking at paediatric anaesthetic care, so it seems a that this cannot be commented on accurately.**

**REPLY**

We thank the reviewer for this comment. We have edited the mentioned sentence, so it is clear we are discussing pediatric anesthetic care across the entire manuscript.

**16. Line 182 – only 7 ventilators in the whole country and Line 256 that 80% of operating rooms are without ventilators. I think it needs to be clarified whether we are looking at a mixture of ventilators (stand alone) and/or ventilators as a part of the anaesthetic machine. In table 2 they are highlighted as a separate entity to the anaesthetic machine. There may be some instances with a stand alone ventilator e.g. icu as opposed to an anaesthetic machine ventilator. It would be good to be clear on whether we are looking at functioning ventilators as a part of the anaesthetic machine delivery.**

**REPLY**

We thank the reviewer for this clarification question. We unfortunately do not have additional data to identify whether we are looking at a mixture of ventilators (stand-alone) and/or ventilators as a part of the anesthetic machine. This has been added as a limitation in the manuscript.

**17. Line 274 – what relevance is full or part-time employment?**

**REPLY**

We thank the reviewer for this question. Full-time and part-time employment are useful information to determine whether personnel worked at multiple hospitals. If so, the number of anesthesia providers by hospital might inflated. Part-time work would also highlight discontinued access to anesthesia care in some hospitals. This explanation has been added to the limitations section of the study.

**18. Line 268 – 50 trained with SAFE courses – these have only been run since 2022-3 thus though good to highlight how things are changing, it wasn’t relevant at that time or needs to be highlighted as something that’s been instituted since the data was collected. Which then should be underlined in line 287 which alludes to the need to deliver SAFE courses regionally – maybe should state that this has already started?**

**REPLY**

We thank the reviewer for this suggestion. We have updated the mentioned sentences to highlight that the SAFE training occurred after the data was collected.

**19. Limitations: I think it is a really unfortunate part of the study that the actual numbers of anaesthetic providers at this time isn’t clear. By own admission there may be cross site working and the number recorded here might be inflated.**

**REPLY**

We agree with the reviewer. We have discussed about these limitations under the limitations section.
